# Data on the effect of improved TiO_2_/FTO interface and Ni(OH)_2_ cocatalyst on the photoelectrochemical performances and stability of CdS cased ZnIn_2_S_4_/TiO_2_ heterojunction

**DOI:** 10.1016/j.dib.2018.01.084

**Published:** 2018-02-06

**Authors:** Mahadeo A. Mahadik, Pravin S. Shinde, Hyun Hwi Lee, Min Cho, Jum Suk Jang

**Affiliations:** a Division of Biotechnology, Advanced Institute of Environmental and Bioscience, College of Environmental and Bioresource Sciences, Chonbuk National University, Iksan 570-752, Republic of Korea; b Pohang Accelerator Laboratory (PAL), Pohang University of Science and Technology POSTECH), Pohang 790-784, Republic of Korea

**Keywords:** Annealed TiO_2_ nanorods, CdS/ZnIn_2_S_4_/TiO_2_ heterostructure, Ni(OH)_2_ cocatalyst, TiO_2_-FTO interface

## Abstract

This data article presents the experimental evidences of the effect of TiO_2_-fluorine doped tin oxide interface annealing and Ni(OH)_2_ cocatalysts on the photoelectrochemical, structural, morphological and optical properties of Ni(OH)_2_/CdS/ZnIn_2_S_4_/TiO_2_ heterojunction. The Raman spectroscopy exhibits the sharp features of the rutile phase of TiO_2_ and in agreement with the X-ray diffraction data. The band gap energy of the 500 °C sample was found to be 3.12 eV, further it was increased to 3.20, 3.22 eV for samples annealed at 600 and 700 °C respectively. The decrease in the band gap energy at 500 °C related to the oxygen vacancies and was analysed by photoluminescence spectroscopy analysis. The synthesis, characterization methods and other experimental details of TiO_2_ based heterostructure are also provided. The presence of CdS and ZnIn_2_S_4_ coating on surface of TiO_2_ electrodes providing a high surface area, extended visible absorption and helps to improve the change separation. This data article contains data related to the research article entitled “Highly efficient and stable 3D Ni(OH)_2_/CdS/ZnIn_2_S_4_/TiO_2_ heterojunction under solar light: Effect of an improved TiO_2_/FTO interface and cocatalyst” (Mahadik et al., 2017) [1].

**Specifications Table**

**Table:** 

Subject area	*Physics, Chemistry*
More specific subject area	*Photoelectrochemical H*_*2*_*S splitting*
Type of data	*Table, image, Graph*
How data was acquired	*Potentiostat (COMPACTSTAT.e, Ivium, Netherland) equipped with impedance analyzer, Solar simulator (Abet Technologies), Field Emission Scanning Electron Microscope (SUPRA 40VP, Carl Zeiss, Germany), Nanofinder 30 spectrometer (Tokyo Inst. Co.) equipped with solid state diode laser (λ*_*ο*_*= 488* *nm) laser, Thermo Scientific XPS spectrometer, Dual beam UV–vis–NIR spectrophotometer (Shimadzu, UV-2600 series, photoluminescence (F-4500 FL spectrophotometer using a Xe arc lamp as the excitation source*
Data format	*Analyzed*
Experimental factors	*J–V measurements of FTO annealed from 400 to 700* *°C, Co-relation between Raman, PL and UV–vis absorptions behaviours with J–V measurements of TiO*_*2*_*/FTO electrodes, Effect of Ni(OH)*_*2*_*on CdS for improving photostability performance.*
Experimental features	*Optimization of TiO*_*2*_*annealing temperature and Ni(OH)*_*2*_*concentration for improve the interface and stability.*
Data source location	*Division of Biotechnology, Advanced Institute of Environmental and Bioscience, College of Environmental and Bioresource Sciences, Chonbuk National University, Iksan, Republic of Korea*
Data accessibility	*Data are available within this article and are related to*[1]

**Value of the data**

• Annealing of TiO_2_-fluorine doped tin oxide (TiO_2_–FTO) interface at various temperatures from 400 to 700 °C, improve the interface and is inhibit the charge recombination.
• Origin of the apparent improvement in the photoelectrochemical (PEC) performance of the annealed TiO_2_ photoelectrodes studied by *J–V* experiments with FTO annealed at 400, 500, 600 and 700 °C.
• The enhancement in stability of CdS/ZnIn_2_S_4_/TiO_2_ is achieved with Ni(OH)_2_ co-catalysts by surface modification and efficient interfacial charge transport between electrolyte and the CdS/ZnIn_2_S_4_/TiO_2_ heterostructure.
• The data are valuable for the nanostructure synthesis and for interfacial charge transfer processes, which can be co-related to the photocurrent behaviour of photoanodes.

## 1 Data experimental design, materials and methods

The data on the heterostructure synthesis and stability obtained using various analytical techniques. In the following sections describes a detailed synthesis of TiO_2_ nanorod based metal sulfides photoanode for efficient photoelectrochemical hydrogen generation. Based on the photocurrent performance, the optimization of annealing temperature of TiO_2_ photoanodes was carried out and used as base materials for fabrication of CdS/ZnIn_2_S_4_/TiO_2_ heterostructure. Further, the effect of a thin layered Ni(OH)_2_ co-catalyst was examined on the stability of the CdS/ZnIn_2_S_4_/TiO_2_ photoelectrode under visible-light irradiation. Finally, the photocurrent responses of all fabricated samples were co-related optical absorption, electrochemical impedance (EIS) and morphological data.

### 1.1 Substrate cleaning

Transparent conducting glass (fluorine-doped tin oxide, FTO, 10–15 Ω cm^−1^) substrates were cut into a required dimension (1 cm × 2.5 cm). The cut substrates were successively cleaned with acetone, ethanol and deionized water in an ultrasonic bath for 10 min each. Finally, the cleaned substrates were dried under low nitrogen (N_2_) stream and used subsequently for hydrothermal deposition of TiO_2_ based electrodes.

### 1.2 Materials and methods

The titanium (IV) butoxide, hydrochloric acid, zinc sulfate heptahydrate, Indium(III) chloride tetrahydrate, thioacetamide, and Cadmium nitrate tetrahydrate, ammonia solution, thiourea, nickel(II) nitrate hexahydrate solution, sodium sulfide nonahydrate, sodium sulfite were analytical reagents and used as received without further purification. Firstly, TiO_2_ nanorods were synthesized on FTO by a facile hydrothermal process according to the literature as reported previously [2]. Briefly, in a typical experiment, the 30 ml deionized water was mixed with 30 ml concentrated hydrochloric acid (36% by weight) and stirred for 5 min. 1 ml titanium butoxide was added into the this solution under constant magnetic stirring for 30 min. The resulting solution was added in a Teflon-lined stainless steel autoclave containing two pieces of FTO substrate on specially designed substrate holder. The hydrothermal synthesis was conducted at 150 °C for 4 h in an electric oven. The as-grown TiO_2_ films were rinsed several times with deionized water and dried in air. As-grown films were annealed at various temperatures from 400 to 700 °C, respectively.

### 1.3 Fabrication of Ni(OH)_2_/CdS/ZnIn_2_S_4_/TiO_2_ architecture

The ZnIn_2_S_4_/TiO_2_ was deposited by adding the 1:2:4 ratios of zinc sulfate heptahydrate, indium (III) chloride tetrahydrate, and thioacetamide (TAA) into 50 ml of distilled water followed by 5 min ultrasonication and then 30 min stirring. After that, the solution was transferred into a Teflon-lined stainless steel autoclave containing TiO_2_ nanorod array photoanodes. The reaction was performed at 160 °C for 2 h. After cooling, the samples were rinsed with ethanol, and dried at room temperature [3]. In the next step, CdS/ZnIn_2_S_4_/TiO_2_/FTO was prepared by adding a 10 mM cadmium nitrate tetrahydrate prepared in the 1 M ammonia solution and 50 mM of thiourea. The resulting solution was transferred into 20 ml glass vials which contain ZnIn_2_S_4_/TiO_2_/FTO glass substrates. The vials were sealed, maintained at 85 °C for 1 h. Finally, Ni(OH)_2_ was deposited on CdS/ZnIn_2_S_4_/TiO_2_ according to a procedure reported previously [4]. In a typical immersion deposition synthesis procedure, various concentrations (5 mM, 10 mM, 15 mM, 25 mM) of nickel(II) nitrate hexahydrate solutions are made in 0.2 M NaOH aqueous medium and CdS/ZnIn_2_S_4_/TiO_2_ electrodes were immersed for 1 h.

## 2 Characterization of Ni(OH)_2_/CdS/ZnIn_2_S_4_/TiO_2_ photoanodes

The Ni(OH)_2_/CdS/ZnIn_2_S_4_/TiO_2_ photoanodes were fabricated by two-step hydrothermal and immersion deposition method. The prepared photoanodes were characterized further to their photoelectrochemical properties, electrochemical impedance spectroscopy, X-ray photoelectron spectroscopy (XPS) using a Nanofinder 30 spectrometer (Tokyo Inst. Co.) equipped with solid state diode laser (*λ*_ο_ = 488 nm) laser, Thermo Scientific XPS spectrometer, surface morphology of the deposited films were examined using a field emission scanning electron microscope (FE-SEM) (SUPRA 40VP, Carl Zeiss, Germany) equipped with an X-ray energy dispersive spectrometer (EDS). Raman spectroscopy was performed to analyse the detailed structural properties. Raman spectra of all the samples were recorded using a Model LCM-S-111 with the excitations from a diode-pumped solid-state (DPSS) 532 nm laser source. The 150 l/mm gratings – detector: CCD detector was used. UV–Vis–DRS spectroscopy (Shimadzu, UV–2600 series) was used in the wavelength range of 300–800 nm. Room temperature photoluminescence (PL) spectra of the samples were measured using an F-4500 FL spectrophotometer with Xe arc lamp as the excitation source.

### 2.1 Photoelectrochemical (PEC) characteristics

**Fig. 1:**
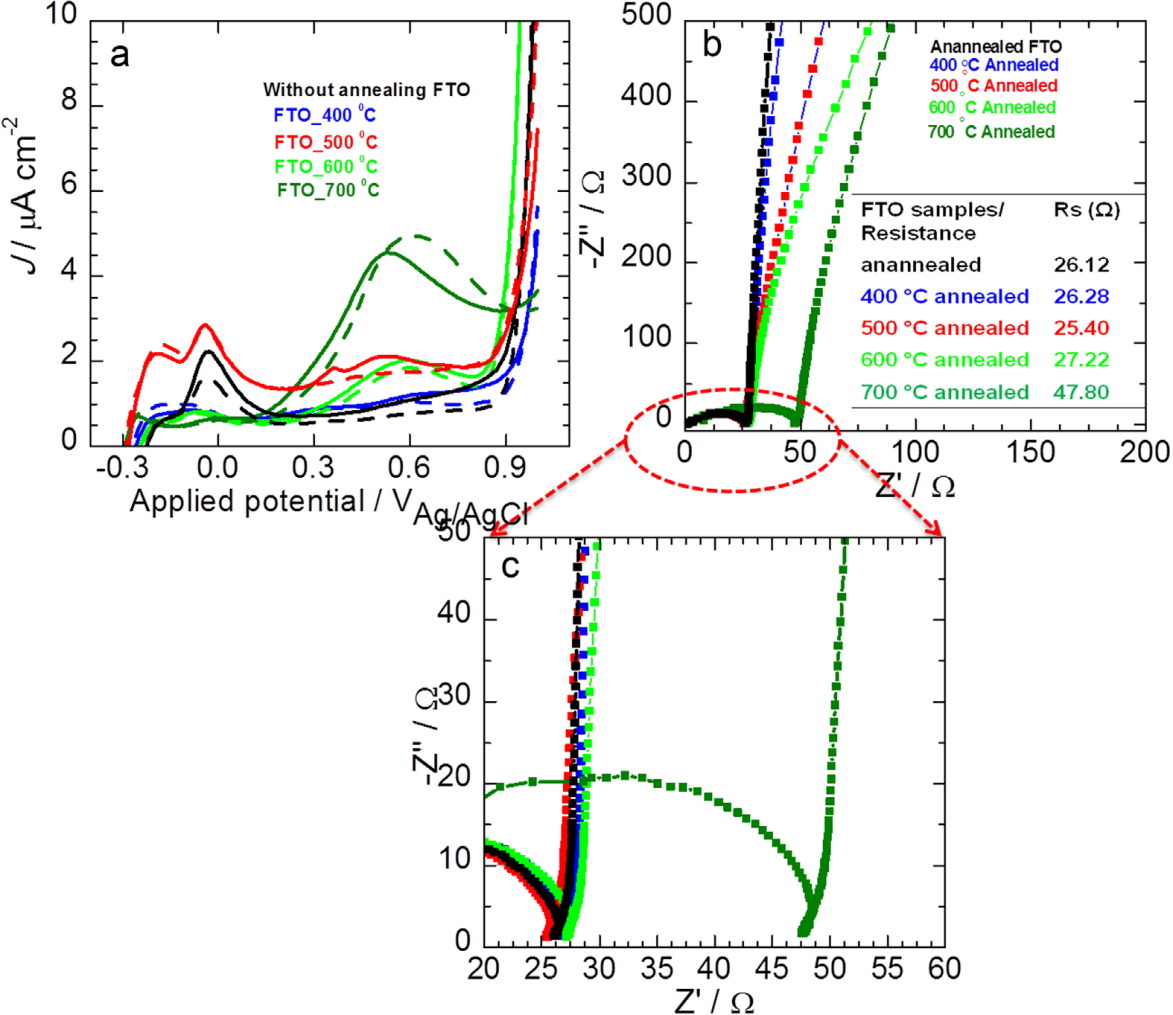
(a) Photocurrent density-potential (*J–V*) characteristics of FTO unannealed and annealed at 400 °C, 500 °C, 600 °C and 700 °C for 1 h (dotted line: dark current; solid lines: light current); (b) Nyquist plots of annealed FTO at 0.1 V vs. Ag/AgCl in the 0.2 M Na_2_SO_4_ electrolyte, inset sheet resistance values at various annealed temperatures; and (c) enlarged part of the high frequency region of EIS spectra.

Optimization of annealing temperature of TiO_2_ was carried out using the photocurrent measurements (*J–V*) with a PEC reactor consisting of three-arm glass compartment with a circular quartz window for light illumination. The electrochemical cell consists of TiO_2_/FTO based photoanodes as working electrode, Pt wire and Ag/AgCl (saturated KCl) were used as the counter and reference electrodes, respectively [5]. The relatively maximum photocurrent density (*J*_ph_) of 988 μA cm^−2^ at 0.1 V vs. Ag/AgCl is observed for sample annealed at 500 °C. Further, to determine the origin of the apparent improvement in PEC of the annealed TiO_2_ photoelectrode, the possible blank (*J–V*) experiments were performed with FTO annealed at (400 °C, 500 °C, 600 °C and 700 °C). No substantial increase in photocurrent nor OCP was observed in FTO electrodes, demonstrating the photocurrent is from TiO_2_ only (Fig. 1a). It has been reported that the, efficiency of most semiconductor photocatalysts is determined to a large degree by recombination rates and the electron transport efficiency [6]. Hence, the electrochemical impedance spectroscopy (EIS) analysis carried out to find out the effect of annealing temperature on the charge transfer resistance of TiO_2_ and correlated with *J*–*V* behaviour. The EIS parameters were determined by fitting the impedance spectra using simple equivalent circuit. In the EIS Nyquist plots, the resistances *R*_f_, and *R*_ct_ are related to the charge transfer resistances of the internal photoanode film as well as photoanode/FTO interface, and at the working electrode/electrolyte interface. The *C*_CPE1_ and *C*_CPE2_: constant phase element of capacitance corresponding to *R*_f_ and *R*_ct_, respectively.

**Fig. 2:**
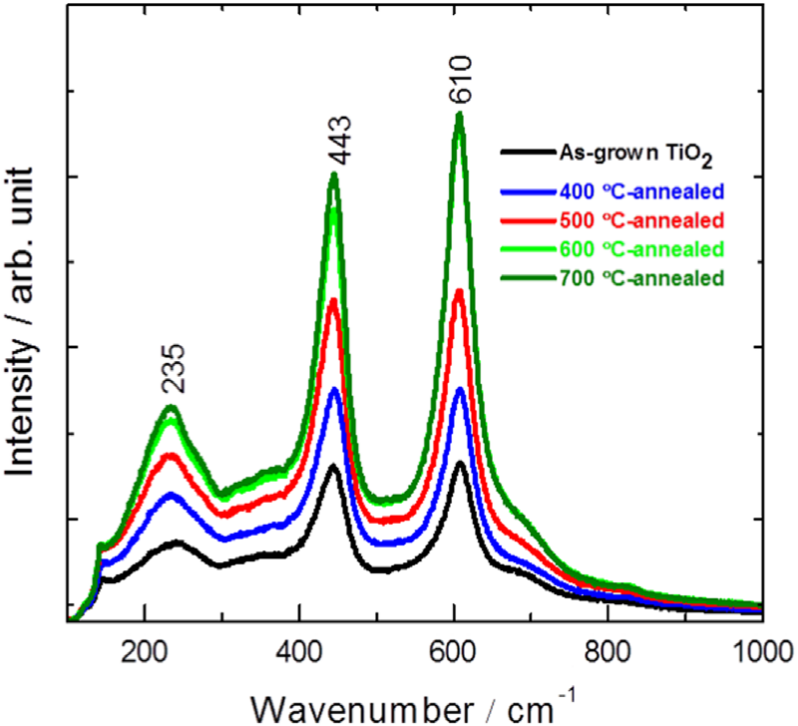
Raman spectra of as-grown and annealed TiO_2_ nanorod photoanods annealed at different temperatures.

It can be inferred that as annealing temperature increases, it is possible to achieve the transformation of the TiO_2_ from amorphous to crystalline. Further, raising the annealing temperature up to 500 °C, resulted in the additional decrease in *R*_f_ and *R*_ct_ because of the improved crystallinity of the TiO_2_ [7]. The decreased *R*_f_ value from 318 to 238 Ω indicating the enhanced contact between FTO and TiO_2_ nanorods interface. Also the charge transfer resistance of the TiO_2_ electrode/electrolyte decreases almost less than half (139–66 Ω), which means the higher annealing temperature would be helpful in suppressing the recombination occurring at the electrode/electrolyte interface. To examine the origin of high photocurrent obtained for 500 °C annealed sample, the photocurrent and EIS study of FTO electrodes annealed at different temperatures were carried out separately (Fig. 1b). The Nyquist plots (real vs. imaginary impedance) of unannealed and annealed FTO electrodes at 400 °C, 500 °C, 600 °C and 700 °C temperatures were measured at a bias potential of 0.1 V vs. Ag/AgCl under 1 sun illumination. The series resistance (*R*_s_), which describes the total resistance from the contributions of FTO, electrical contacts, etc., was determined and shown in the table (inset of Fig. 1b). The unannealed and annealed FTO shows relatively similar values of *R*_s_ up to 600 °C annealed temperature, however, at 700 °C shows higher resistance due to the Sn diffusion [8]. Thus, the photocurrent density in annealed TiO_2_/FTO samples comes from the TiO_2_ only and not from the FTO. To study the influences of annealing temperature on the structure of annealed TiO_2_ samples, the Raman spectra were measured and are shown in Fig. 2. The Raman modes at 235, 443, and 610 cm^−1^ are coming from the rutile phase of TiO_2_. These band positions are in accordance with the values reported in the previous literature studies for the rutile [9]. It is seen that the intensity of these Raman peaks appears to increase with increasing temperatures. The peak position and broadening of the Raman spectrum is mainly affected by the size of the nanomaterial as well as defects [10]. The broad peak observed near 160–240 cm^−1^ is assigned to O–O interactions involving three- and four-coordinate oxygen. These peaks are initially observed for as-grown samples and become sharper and more intense as the annealing temperature is increased from 400 to 700 °C, indicating increased crystallinity of the rutile phase.

The red shift of *E*_g_ (443 cm^−1^) and A_1g_ (610 cm^−1^) modes is generally due to the phonon confinement effect and oxygen vacancy defects in rutile TiO_2_ [11]. Consequently, the ratio of these integrated Raman peaks, *I*_443_/*I*_610_, serves as a measure of the degree of crystallinity for rutile. For the as-grown TiO_2_, the results show very low photocurrent densities of 40 µA cm^−2^; this corresponds to less crystallinity and defects present in the sample. The photocurrent density increases up to 988 µA cm^−2^ as the annealing temperature increases from 400 to 500 °C, with the increases in crystallinity. Thus, as the annealing temperature is increased, the amount and crystallinity of the rutile increase and so does the photo-activity [12].

**Fig. 3:**
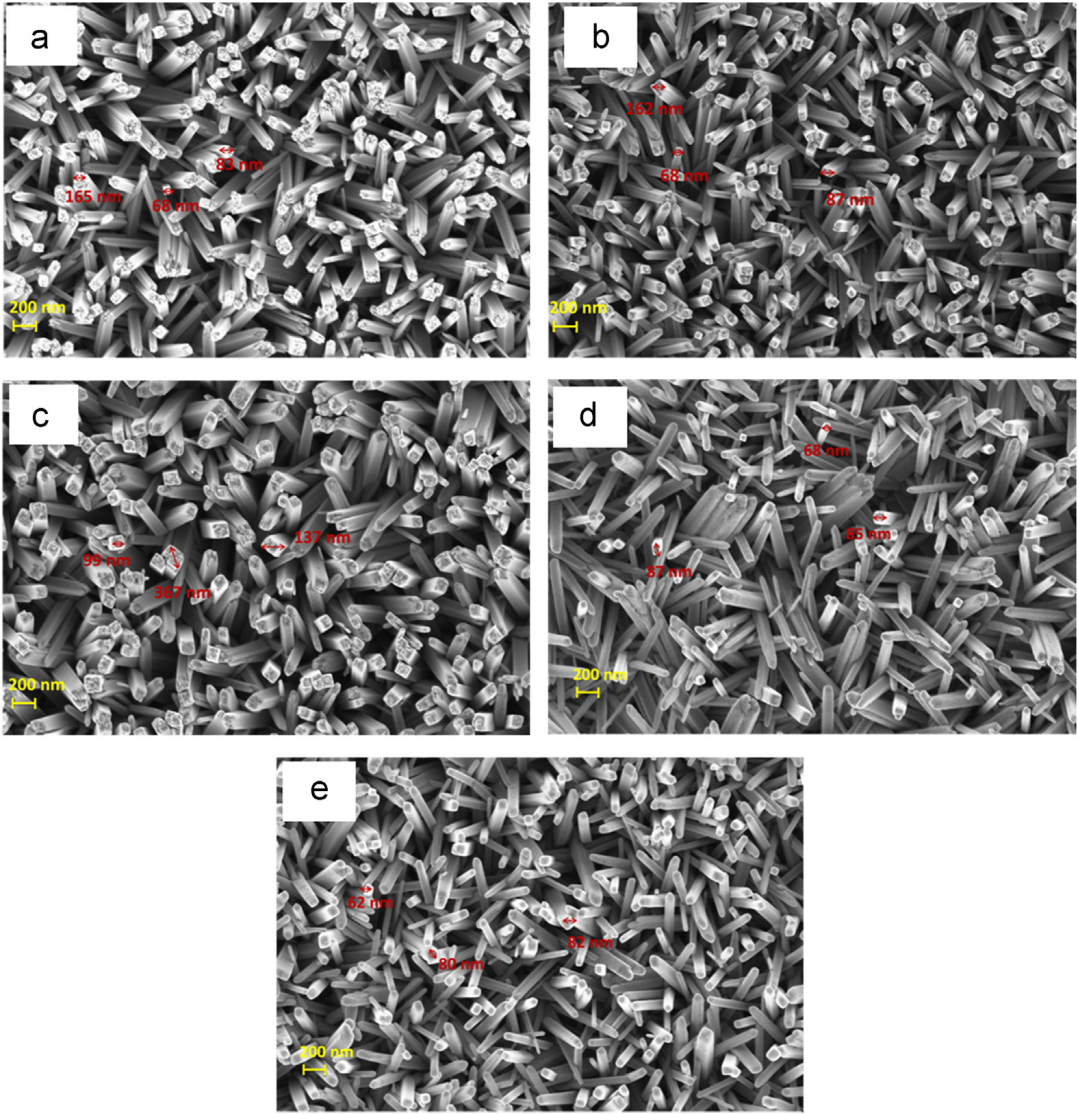
FE-SEM images of (a) as-grown TiO_2_ films, annealed at: (b) 400 °C, (c) 500 °C, (d) 600 °C, and (e) 700 °C for 1 h.

However, above 500 °C, it was seen that, even though there is an increase in crystallinity of rutile, the photocurrent density was decreased. This is due to increased grain size (as seen from cross-sectional FE-SEM) at the TiO_2_/FTO interface which gives enhancement in the grain boundaries and results in higher resistance in accordance with the EIS data Table 1. Thus, an increase in the annealing temperature removes the defect levels at 500 °C. However, at higher temperatures (i.e. 600 and 700 °C), the photoelectrochemical response was again decreased, suggesting that the photo-response is significantly affected by some other superseding factor in addition to crystallinity. During the annealing process, ruptures of grain boundary between the growing and shrinking grains, results in detachment of atoms which leads to the reduction in the photocurrent at higher temperatures. To study the effect of heating process on the size of TiO_2_ nanorods, the top view FE-SEM images of as-grown and annealed TiO_2_ at different temperatures (400–700 °C) are shown in Fig. 3. It is seen that as-grown and 400 °C annealed samples shows approximately same size diameter of TiO_2_ nanorods (83–165 nm). However, the main difference is that as-grown samples shows rough surface of TiO_2_, whereas in 400 °C annealed samples TiO_2_ surface becomes smooth. Further, increase in the annealing temperature up to 500 °C, the diameter of nanorods increased again with the smoother surface. The average diameter of TiO_2_ nanorods at 500 °C annealing temperature is approximately 99–367 nm. However, for the samples annealed at 600 and 700 °C, the diameter of nanorods is decreased to 68–87 nm and 62–80 nm respectively for 600 °C and 700 °C annealing temperatures. It is also revealed that the TiO_2_ nanorods uniformly covered the surface of the substrate and are nearly perpendicular to the FTO substrate, in agreement with the XRD results.

**Table 1:** EIS fitted parameters of as-grown and annealed TiO_2_ photoelectrodes.

TiO_2_ samples/EIS parameters	*R*_s_	*R*_f_	*R*_ct_	*C*_CPE1_	*C*_CPE2_
	Ω	Ω	Ω	µF	μF
As-grown	28	34,933	2425	13	39
400 °C-annealed	31	318	139	306	18
500 °C-annealed	47	238	66	767	718
600 °C-annealed	29	595	448	3.5	153
700 °C-annealed	50	681	1670	2.1	105

**Fig. 4:**
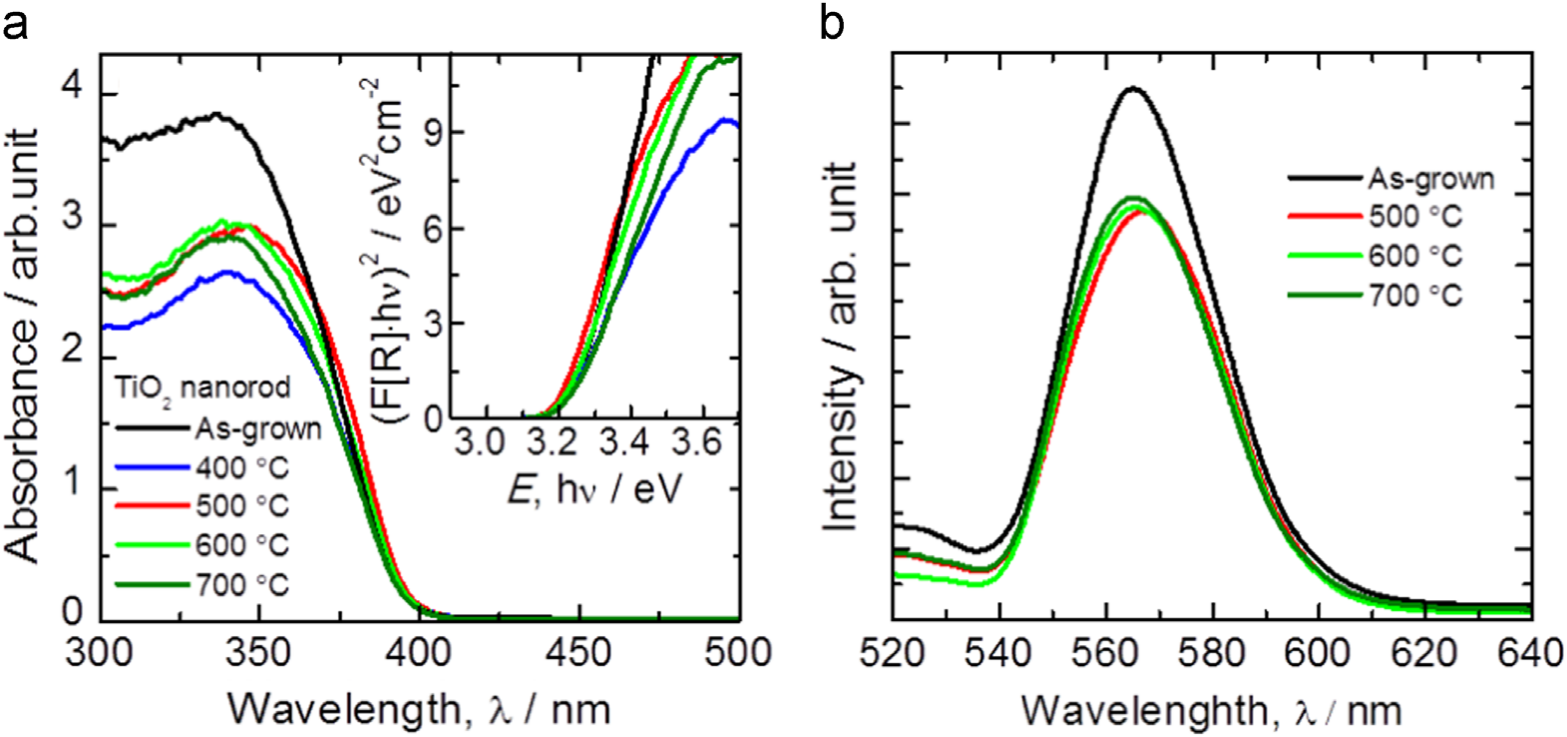
(a) UV–vis absorbance and Tauc plots (inset) and, (b) photoluminescence spectra of as-grown and annealed TiO_2_ films at different temperatures for 1 h.

The UV–vis absorbance spectra of as-grown and annealed TiO_2_ films were recorded in the wavelength range of 350–500 nm and are shown in Fig. 4a. The band-gap energy (*E*_g_) values of the as-grown and annealed TiO_2_ were calculated using the well-known Tauc's plot method and are shown in the inset of Fig. 4a. The calculated *E*_g_ values of as-grown and TiO_2_ annealed at 400 °C, 500 °C, 600 °C, 700 °C temperatures are 3.25, 3.21, 3.12, 3.20 and 3.22 eV, respectively. The reduction in the *E*_g_ value of TiO_2_ annealed at 500 °C is due to quantum size effect. However, the band gap variation with TiO_2_ diameter is also a well-known phenomenon in the case of II–IV semiconductor nanocrystals [13], [14]. As photoluminescence (PL) emissions result from the recombination of free carriers, the PL is another suitable tool to study the efficiency of charge carrier trapping, migration, transfer, and to understand the fate of electron–hole pairs in deposited TiO_2_ photoanodes. The recombination of photoinduced electrons and holes releases energy in the form of PL emission spectra. The PL peaks located in the visible luminescence band are correlated to oxygen vacancies on the surface of TiO_2_ and that the intensity of the emission peaks increases with the defect levels [15], [16]. Fig. 4b shows the higher PL intensity for as-grown TiO_2_ photoanode than that of the annealed one. This is due to the rapid recombination of photoinduced charge carriers in the as-grown samples. However, the PL intensity is again increased at 700 °C annealed temperatures compared to 500 °C, due to the TiO_2_ thin film with better crystallization. Thus, the variation in PL spectra of TiO_2_ photoanodes is due to different surface microstructures of the annealed samples [17].

**Fig. 5:**
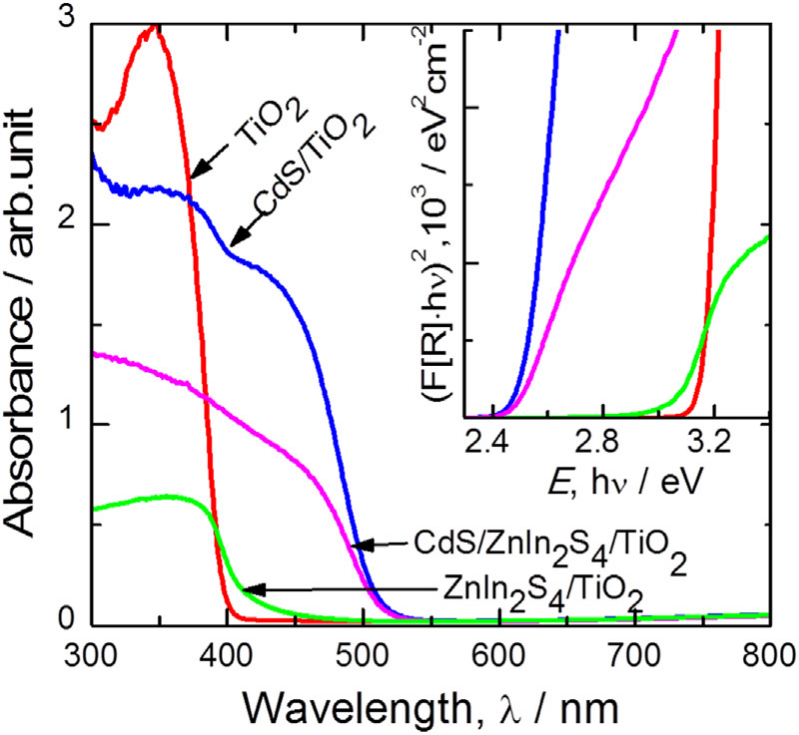
UV–vis absorbance and Tauc plots (inset) for annealed TiO_2_, CdS/TiO_2_, ZnIn_2_S_4_/TiO_2_ and CdS/ZnIn_2_S_4_/TiO_2_ thin films.

To confirm the growth of ZnIn_2_S_4_ and CdS on annealed TiO_2_ and the band gap energy for the electrodes, UV–vis absorbance spectra were measured and the results are shown in Fig. 5. The UV–vis absorption spectra of CdS/ZnIn_2_S_4_/TiO_2_ and CdS/TiO_2_ electrodes are extended to visible region compared to that of pristine TiO_2_ and ZnIn_2_S_4_/TiO_2_. However, the small shift in the visible region was observed for the CdS/ZnIn_2_S_4_/TiO_2_ electrode as compared to the CdS/TiO_2_ electrode, mostly due to the small thickness of ZnIn_2_S_4_. Thus the improved photoelectrochemical performance of CdS/ZnIn_2_S_4_/TiO_2_ was supplemented by the absorbance spectra of the material, which showed enhanced visible light absorbance and a reduction in the band gap from 3.0 eV to 2.5 eV for pristine TiO_2_ to CdS/ZnIn_2_S_4_/TiO_2_ (inset of Fig. 5).

**Fig. 6:**
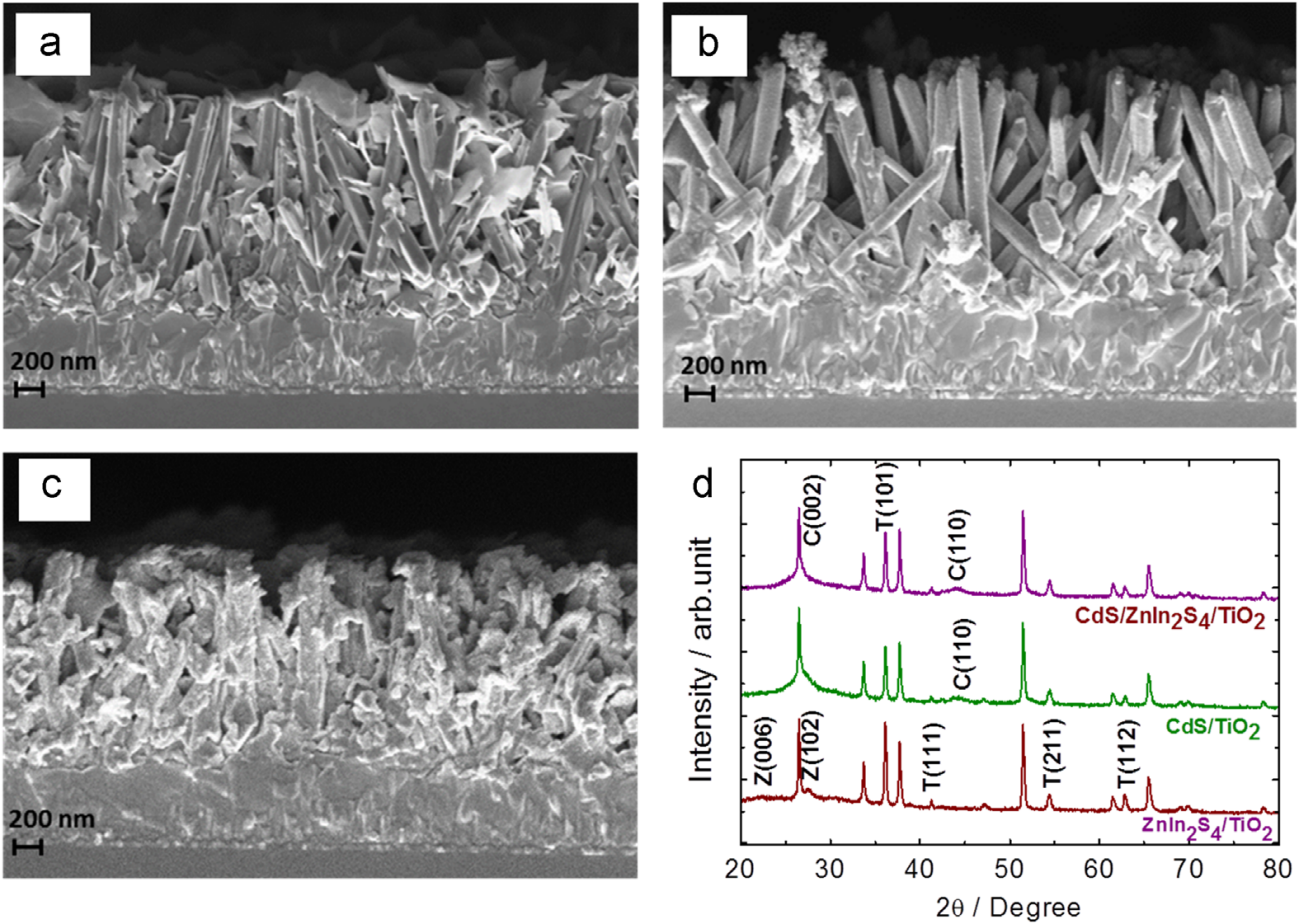
(a-c) FE-SEM cross-sectional views, and (d) XRD patterns of ZnIn_2_S_4_, CdS, CdS/ZnIn_2_S_4_ on annealed TiO_2_ nanorod arrays.

Fig. 6a depicts that the TiO_2_ nanorod array is covered by a ZnIn_2_S_4_ network of interconnected nanosheets, providing a high surface area, which have potential applications in the areas of catalysis and energy conversion [18]. The FE-SEM images of CdS/TiO_2_ (Fig. 6b) also shows that the surfaces of the nanorods became rough after coating, confirming that the surface coating of the TiO_2_ nanorods with CdS nanoparticles. Thus, due to CdS coatings the diameters of TiO_2_ nanorod arrays are increased from 97 nm to 120 nm. However, The FE-SEM top view of CdS/ZnIn_2_S_4_/TiO_2_ indicates the small amount of CdS nanograins incorporates into the network of ZnIn_2_S_4_/TiO_2_ and majority of CdS nanograins remains on the surface of ZnIn_2_S_4_ nanosheet giving a higher thickness of photoelectrode providing a high surface area.

The cross-sectional image of CdS/ZnIn_2_S_4_/TiO_2_ (Fig. 6c) demonstrates that the gap between TiO_2_ nanorods almost disappears due to insertion of the ZnIn_2_S_4_ nanosheets and CdS nanograins. This helps to improves the light absorption and charge separation in the photoanode. In addition to this, to identify the relationship between electrochemical performance, charge transfer, and recombination in TiO_2_-based photoelectrodes, the impedances of TiO_2_-based electrodes were measured and results are depicted in Table 2. Fig. 6d shows the XRD patterns of as-grown ZnIn_2_S_4_/TiO_2_, CdS/TiO_2_, CdS/ZnIn_2_S_4_/TiO_2_ heterostructure arrays. In case of the XRD pattern of ZnIn_2_S_4_/TiO_2_/FTO, the diffraction peaks (i.e. Z(006) and Z(102)) other than FTO and TiO_2_ (JCPDS#21-1272) are attributed to the hexagonal phase of ZnIn_2_S_4_ space group: *P*63*mc*, *a* = *b =* 3.85(2) Å, *c* = 24.68(4) Å, JCPDS 72-0773) [19], [20]. The XRD pattern of CdS/TiO_2_/FTO indicates the peaks at 2*θ* = 26.5 and 44.5 corresponded to the diffractions of the (002) and (110) plane of hexagonal wurtzite phase of CdS (JCPDS 89-2944) and are termed as C(002), C(110).

**Table 2:** EIS fitting parameters of TiO_2_ annealed at 500 °C, ZnIn_2_S_4_/TiO_2_, CdS/TiO_2_ and CdS/ZnIn_2_S_4_/TiO_2_ photoelectrodes.

Samples/parameters	*R*_s_	*R*_f_	*R*_ct_	*C*_*CPE1*_	*C*_CPE2_
	Ω	Ω	Ω	μF	μF
TiO_2_ annealed	47	238	66	767	718
ZnIn_2_S_4_/TiO_2_	29	527	79	527	145
CdS/TiO_2_	26	2755	86	12	34
CdS/ZnIn_2_S_4_/TiO_2_	34	723	29	143	2050

However, in case of CdS/ZnIn_2_S_4_/TiO_2_ heterostructure thin film, the suppressed peak intensity of ZnIn_2_S_4_ and TiO_2_ suggesting that CdS is present on the surface of ZnIn_2_S_4_/TiO_2_.

**Fig. 7:**
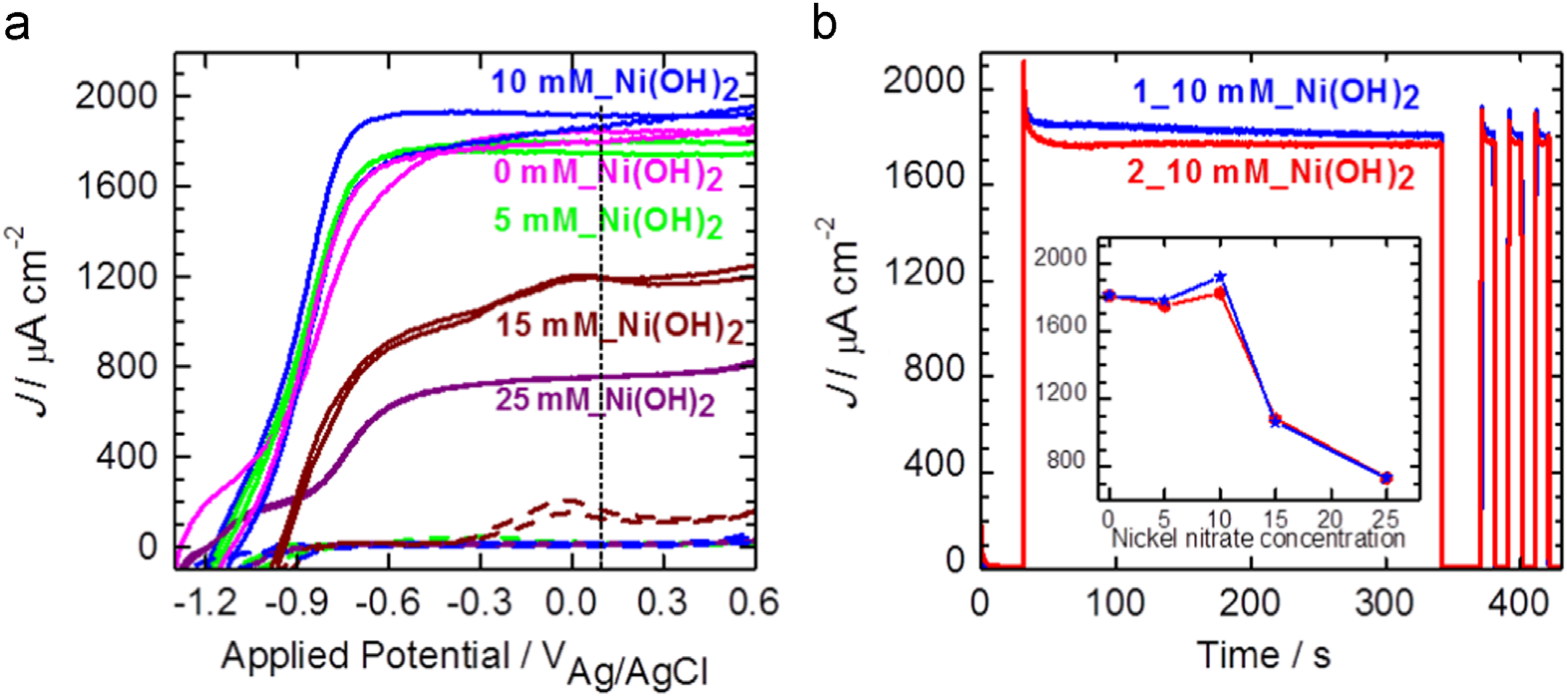
(a) *J–V* plots collected from two identical conditions samples of Ni(OH)_2_ coated CdS/ZnIn_2_S_4_/TiO_2_ photoanodes, (b) photostability and transient photocurrent responses, inset shows the variation of *J*_ph_ vs. Nickel nitrate concentration for Ni(OH)_2_/CdS/ZnIn_2_S_4_/TiO_2_ photoanodes measured at 0.1 V vs. Ag/AgCl.

To study the effect of Ni(OH)_2_ layer on the photoactivity and stability of CdS/ZnIn_2_S_4_/TiO_2_ photoelectrode, the 5, 10, 15, and 25 mM nickel nitrate solutions were prepared and as-synthesized CdS/ZnIn_2_S_4_/TiO_2_ electrodes were dipped in these solutions for 1 h. Fig. 7a shows the current density–voltage (*J–V*) curves of Ni(OH)_2_/CdS/ZnIn_2_S_4_/TiO_2_ measured at 0.1 V vs. Ag/AgCl for different concentration of nickel precursor. *J–V* measurements were carried out for two samples fabricated under identical condition.

It is seen that, for 5 mM and 10 mM nickel nitrate concentration shows the approximately same *J–V* performance as does CdS/ZnIn_2_S_4_/TiO_2_ but it exhibits improved photoelectrode stability (Fig. 7b). Amongst the studied samples 10 mM nickel precursor shows higher photocurrent density (*J*_ph_), therefore, considered as the optimum (inset of Fig. 7b). In order to achieve more stability of photoelectrodes, the intimate contact between the co-catalyst and the CdS/ZnIn_2_S_4_/TiO_2_ heterostructure is an important factor for efficient charge separation and transfer; hence, to study this factor, impedance measurements with EIS were carried out and results shown in Table 3. It is seen that 10 mM nickel nitrate concentration shows less values of *R*_f_ and *R*_ct_, and thus responsible for the higher photocurrent density.

**Table 3:** EIS fitted parameters of the Ni(OH)_2_/CdS/ZnIn_2_S_4_/TiO_2_ heterostructure at various nickel nitrate concentrations.

Concentrations of nickel nitrate/EIS parameters	*R*_s_	*R*_f_	*R*_ct_	*C*_*CPE1*_	*C*_CPE2_
	Ω	Ω	Ω	μF	μF
5 mM	70	588	74	303	100
10 mM	46	357	76	403	0.5
15 mM	39	674	71	305	101
25 mM	42	1299	203	195	0.43

**Fig. 8:**
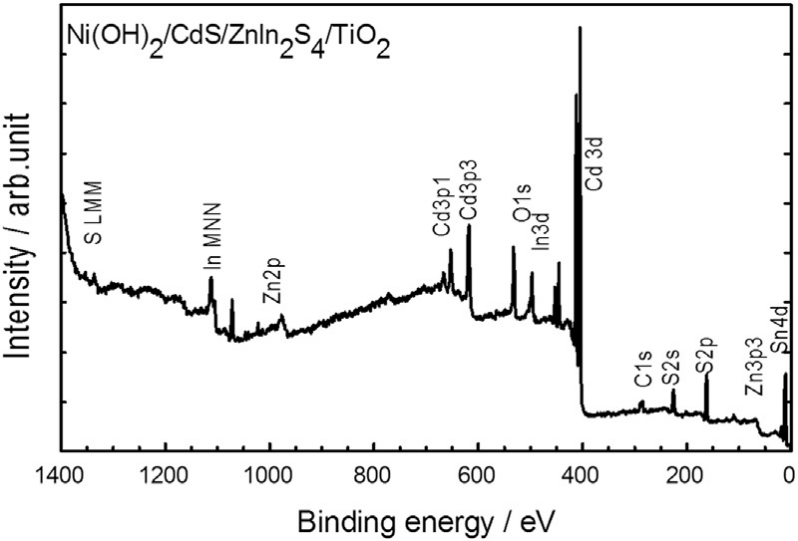
XPS survey spectrum of 10 mM Ni(OH)_2_ on CdS/ZnIn_2_S_4_ prepared on 500 °C annealed TiO_2_.

The chemical state and elemental quantification in the Ni(OH)_2_/CdS/ZnIn_2_S_4_/TiO_2_ thin film were performed using XPS equipped with a monochromatic Al Kα X-ray source (*hν* = 1486.6 eV). Fig. 8 shows the XPS survey scan spectra of Ni(OH)_2_/CdS/ZnIn_2_S_4_/TiO_2_ fabricated by dip coated and double hydrothermal methods. The binding energy (BE) peaks centered at 411.4, 404.7, 1021.67, 1045.12, 162.61, 161.39, 458.7 and 530.8 eV are assigned to Cd 3d_3/2_, Cd 3d_5/2_, Zn 2p_3/2_, Zn 2p_1/2_, S 2p_1/2_, S 2p_3/2_, Ti 2p_3/2_, and O 1s photo-electrons, respectively. The broad peaks centered at 1011.14, and 1334.14 eV are assigned to the In_MNN_ and S_LMM_ Auger transitions, respectively. Among all the peaks, the Ni 2p peaks at 856.2 eV is very weak and is not observed in the survey scan. This is due to the low thickness of Ni (OH)_2_ on the surface of CdS/ZnIn_2_S_4_/TiO_2_ surface.

**Fig. 9:**
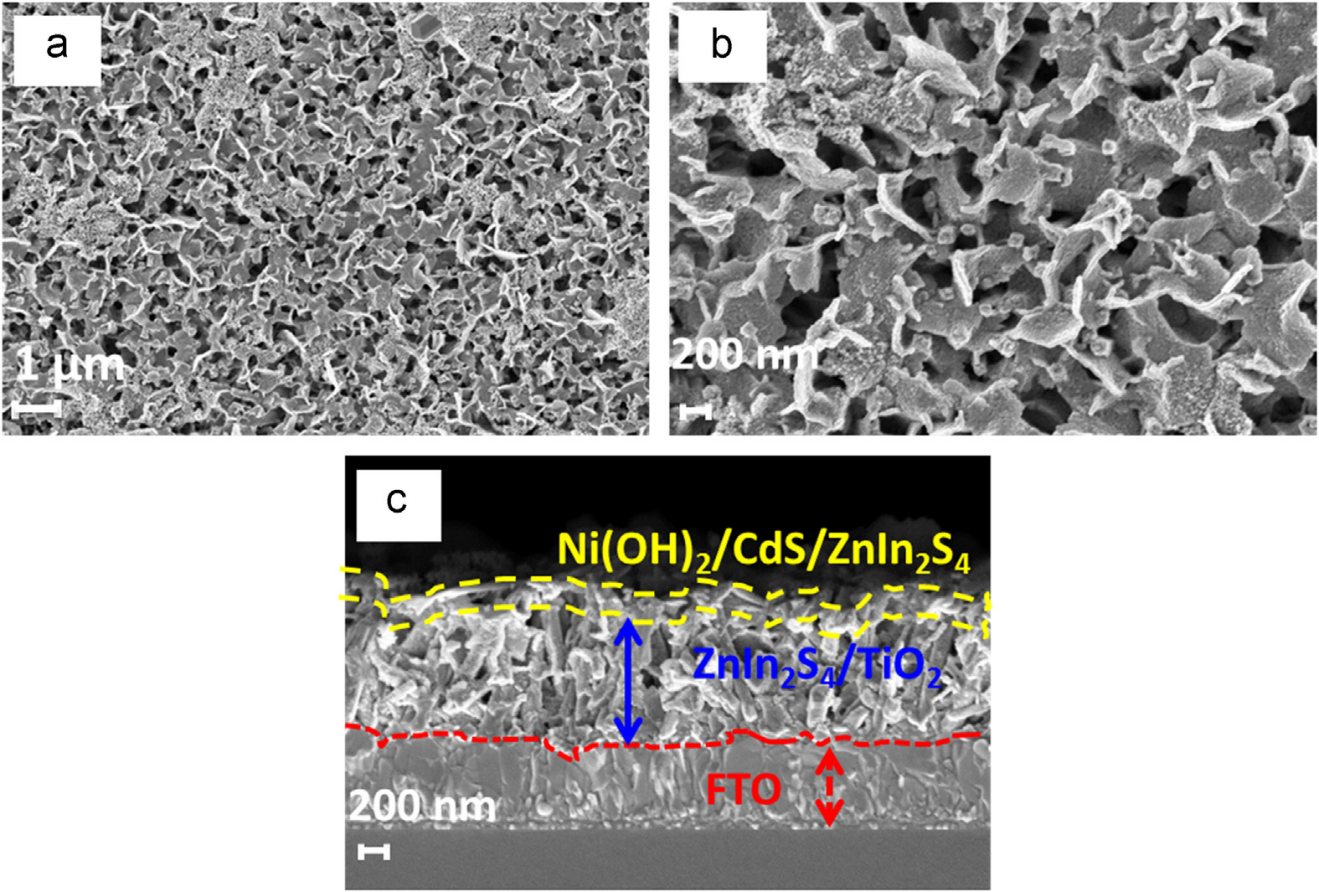
FE-SEM top views (a, b) and (c) cross-sectional view of Ni(OH)_2_/CdS/ZnIn_2_S_4_ on annealed TiO_2_ nanorod arrays.

Fig. 9 shows the typical top view FE-SEM images of the as prepared Ni(OH)_2_/CdS/ZnIn_2_S_4_/TiO_2_/FTO films. As shown in Fig. 9a, the entire surface composed of a uniformly distributed network of interconnected ZnIn_2_S_4_ nanosheets and is covered with CdS and Ni(OH)_2_ nanoparticles. Fig. 9b represents a high magnification FE-SEM image of the Ni(OH)_2_/CdS/ZnIn_2_S_4_/TiO_2_ film, which clearly shows that the ZnIn_2_S_4_/TiO_2_ nanorod arrays covered with non-uniformly distributed Ni(OH)_2_/CdS aggregated nanoparticles. However, the cross sectional FE-SEM image of the Ni(OH)_2_/CdS/ZnIn_2_S_4_/TiO_2_ film shows that some amount of CdS, and Ni(OH)_2_ nanograins is incorporated into the lattice of ZnIn_2_S_4_/TiO_2_ and reduces the empty space in photoanode. The formation of Ni(OH)_2_/CdS coating on the surface of ZnIn_2_S_4_/TiO_2_ (yellow colour line) are shown in Fig. 9(c) and the remaining part up to FTO is ZnIn_2_S_4_/TiO_2_ nanorods (blue arrow). However, using the FE-SEM images, it is quite difficult to confirm the presence of Ni(OH)_2_ on the surface of CdS/ZnIn_2_S_4_/TiO_2_ heterostructure. Hence, the presence of Ni(OH)_2_ and CdS on the surface of ZnIn_2_S_4_/TiO_2_ is also confirmed from the XPS results. It is also seen from the PEC analysis that the increase in concentration of nickel nitrate, the photocurrent starts decreases. This decreased photocurrent contributes due to the increased path resistance of photogenerated charges as shown in Table 3.
