# Supplementary material for: Data on the effect of improved TiO2/FTO interface and Ni(OH)2 cocatalyst on the photoelectrochemical performances and stability of CdS cased ZnIn2S4/TiO2 heterojunction
Source: Data Brief. 2018 Feb 6;17:807–19. doi: 10.1016/j.dib.2018.01.084 (PMC5842242; doi:10.1016/j.dib.2018.01.084)
Supplement: Supplementary file 1 — Transparency document [file mmc1.docx]

*November 12, 2016*

**Cover Letter**

To the Editor,

*Data in Brief*

Dear Sir or Madame:

We are submitting our revised manuscript (Ms. Ref. No.: DIB-D-16-00749R1), entitled “Upgradation in photoelectrochemical performances and stability of CdS cased ZnIn_2_S_4_/TiO_2_ heterojunction via improved TiO_2_/FTO interface and Ni(OH)_2_ cocatalyst” by Mahadeo A. Mahadik, Pravin S. Shinde, Hyun Hwi Lee, Min Cho, and myself for consideration for publication in the Data in Brief - Journal.

We have revised the manuscript substantially in view of the reviewers’ valuable comments, addressed all the reviewers’ points, and incorporated the relevant changes into the revised manuscript. The changes incorporated in the manuscript are highlighted in blue. A word file addressing the answers to the reviewers’ comments has been uploaded separately. All co-authors have approved of the revised manuscript and agree with its contents.

We hope that the revised manuscript addresses all of the queries raised by the reviewers. There is no conflicts of interest.

Thank you very much for considering our manuscript for publication.

Sincerely,

**Prof. Jum Suk Jang**

Division of Biotechnology, Advanced Institute of Environmental and Bioscience,

College of Environmental and Bioresource Sciences, Chonbuk National University,

Iksan 570-752, Republic of Korea

E-mail: [jangjs75@jbnu.ac.kr](mailto:jangjs75@jbnu.ac.kr)
